# Conjugates of Gold Nanoparticles and Antitumor Gold(III) Complexes as a Tool for Their AFM and SERS Detection in Biological Tissue

**DOI:** 10.3390/ijms20246306

**Published:** 2019-12-13

**Authors:** Aleksandra M. Bondžić, Andreja R. Leskovac, Sandra Ž. Petrović, Dragana D. Vasić Anićijević, Marco Luce, Lara Massai, Amanda Generosi, Barbara Paci, Antonio Cricenti, Luigi Messori, Vesna M. Vasić

**Affiliations:** 1 Vinča Institute of Nuclear Sciences, University of Belgrade, P.O. Box 522, 11000 Belgrade, Serbia; aleksandrab@vin.bg.ac.rs (A.M.B.); andreja@vin.bg.ac.rs (A.R.L.); sandra@vin.bg.ac.rs (S.Ž.P.); draganav@vin.bg.ac.rs (D.D.V.A.); 2 Istituto di Struttura della Materia, Consiglio Nazionale delle Ricerche, 00132 Roma, Italy; marco.luce@artov.ism.cnr.it (M.L.); amanda.generosi@ism.cnr.it (A.G.); barbara.paci@ism.rm.cnr.it (B.P.); antonio.cricenti@artov.ism.cnr.it (A.C.); 3 Department of Chemistry, University of Florence, Via della Lastruccia 3, 50019 Sesto Fiorentino, Italy; lara.massai@unifi.it (L.M.); luigi.messori@unifi.it (L.M.)

**Keywords:** gold(III) antitumor complexes, AuNPs, conjugation, cell fractions, AFM, SERS

## Abstract

Citrate-capped gold nanoparticles (AuNPs) were functionalized with three distinct antitumor gold(III) complexes, e.g., [Au(N,N)(OH)_2_][PF_6_], where (N,N)=2,2′-bipyridine; [Au(C,N)(AcO)_2_], where (C,N)=deprotonated 6-(1,1-dimethylbenzyl)-pyridine; [Au(C,N,N)(OH)][PF_6_], where (C,N,N)=deprotonated 6-(1,1-dimethylbenzyl)-2,2′-bipyridine, to assess the chance of tracking their subcellular distribution by atomic force microscopy (AFM), and surface enhanced Raman spectroscopy (SERS) techniques. An extensive physicochemical characterization of the formed conjugates was, thus, carried out by applying a variety of methods (density functional theory—DFT, UV/Vis spectrophotometry, AFM, Raman spectroscopy, and SERS). The resulting gold(III) complexes/AuNPs conjugates turned out to be pretty stable. Interestingly, they exhibited a dramatically increased resonance intensity in the Raman spectra induced by AuNPs. For testing the use of the functionalized AuNPs for biosensing, their distribution in the nuclear, cytosolic, and membrane cell fractions obtained from human lymphocytes was investigated by AFM and SERS. The conjugates were detected in the membrane and nuclear cell fractions but not in the cytosol. The AFM method confirmed that conjugates induced changes in the morphology and nanostructure of the membrane and nuclear fractions. The obtained results point out that the conjugates formed between AuNPs and gold(III) complexes may be used as a tool for tracking metallodrug distribution in the different cell fractions.

## 1. Introduction

Among the large number of gold(III) complexes synthesized so far with the goal to overcome the toxic side effects of platinum(II) drugs in anticancer treatment [1,2], the compounds having aromatic N-containing heterocycles as ligands have gained special interest, as such ligands greatly increase the stability of these metal compounds under physiological conditions [3,4,5,6]. Moreover, N-containing heterocycles and their derivatives as the structural moieties of metal-based drugs confer to the metal center diverse biological activities, such as antitumor, antimicrobial, and anti-inflammatory [4,7]. For this reason, their tracking in biological tissues is of great importance [8,9].

Unlike platinum(II) drugs that produce their antitumor effects by causing direct chemical damage to genomic DNA in cancer cells [10,11], it was found that proteins, rather than DNA, are the main targets for the biological action of gold(III) antitumor complexes [6,11,12]. In addition, independent studies pointed out that the interactions of these complexes with target cellular proteins result in the modification of surface protein residues and loss of protein functions [13]. Some of these enzymes, such as thioredoxin reductase, glutathione transferase, cysteine protease, and ATPases, are overexpressed in cancer cells [13,14,15,16]. Recently, the combined theoretical and experimental studies of the interaction of some mono and binuclear gold(III) complexes containing N-heterocyclic ligands and their derivatives (pyridine, bipyridine) indicated that they inhibit the Na/K pump by interacting with its extracellular part [17,18,19]. However, this enzyme is highly expressed in tumor cells, especially in the plasma membrane of non-neoplastic cells, and mainly in the cytoplasm of tumor cells [16,20].

The identification and localization of drugs inside cells are of vital importance for their medical application as well as for the investigation of their fate and their influence on inner cell organs and components [21,22]. In general, the nanoparticles (NPs) surface and core properties can be manipulated for specific applications such as molecular recognition, chemical sensing, and imaging [23,24,25]. For this purpose, drug-functionalized noble metals NPs (AuNPs, AgNPs) in combination with nanotechnology-based methods (atomic force microscopy (AFM), surface-enhanced Raman spectroscopy (SERS), and fluorescence spectroscopy), may represent an excellent nanoplatform for developing drug delivery and biosensing systems [24,26,27,28,29]. The changes of nanostructural features of the cell membrane, i.e., mean height of particles, root-mean-square roughness (Rq), crack and fragment appearance, were observed in the studies of biophysical properties (morphology, nanostructure, adhesion force, stiffness, and others) of cells near physiological conditions and provided fundamental insights into the cellular structures and biological functions [21,27,30].

**Scheme 1:**
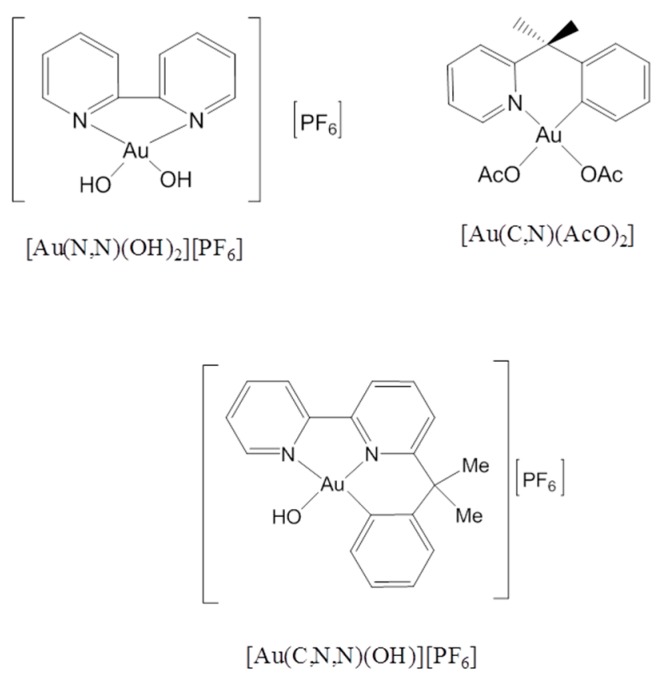
Chemical structures and trivial names of the studied gold(III) complexes.

In this work, we functionalized the citrate capped AuNPs [31,32] by selected antitumor mononuclear gold(III) complexes (Scheme 1), which have bipyridine and pyridine ligands, to study the potential opportunities for their tracking in biological tissues. The formed nanocomposites were characterized by AFM [33], DFT calculation [34,35,36,37,38,39], UV/Vis spectrophotometry, DLS, and Raman spectroscopy methods. The aim of the work was the intracellular tracking of gold(III) complexes’ distribution by forming in situ their AuNPs conjugates. Harvested human lymphocytes were employed as a model system in order to evaluate the distribution of conjugates in cell fractions using SERS and also the changes induced by them in cellular fractions using AFM.

## 2. Results

### 2.1. Prediction of Gold(III) Complexes Binding to Citrate-Capped AuNPs

**Figure 1:**
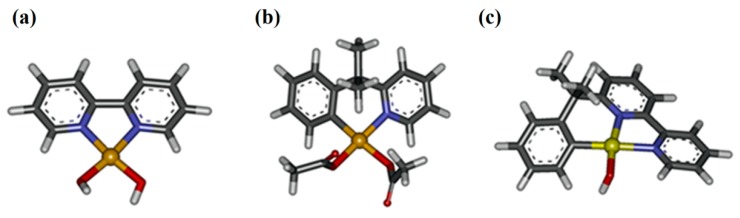
DFT-optimized geometry of gold(III) complexes visualized with the XcrysDen software. (**a**) [Au(N,N)(OH)_2_][PF_6_]; (**b**) [Au(C,N)(AcO)_2_]; (**c**) [Au(C,N,N)(OH)][PF_6_].

The ability of the investigated gold(III) complexes to bind to the pure gold surface was examined by DFT calculations in order to gain better insight into the fundamental interactions between the selected complexes and the core of the AuNPs. The results for the DFT optimization of complex cations ([Au(C,N,N)(OH)]^+^, [Au(N,N)(OH)_2_]^+^) and neutral complex [Au(C,N)(AcO)_2_] geometry revealed a rather planar complex structure (Figure 1) which is in good agreement with their previously obtained structural features [3,40].

Optimized adsorption geometries and the positioning of the complexes on the AuNPs surface, closest complex-surface distances, adsorption energies, and the number of electrons received by the molecule of the complex are given in Table 1.

Negative values of the adsorption energies were obtained for all nanocomposites, implying that the adsorption of the complexes on the Au(Ш) surface is thermodynamically possible. In the case of [Au(N,N)(OH)_2_]^+^, two possible orientations on the AuNPs surface were investigated: parallel (flat-on coordination) and vertical (end-on conformation). The results indicated that the flat-on complex orientation, i.e., the complex cation binding to the NPs indirectly, via partially positive pyridine ring interaction with AuNPs core, is energetically more favorable than the vertical one, which includes the binding to the AuNPs surface via hydroxyl groups of the complex. For the charged complexes [Au(N,N)(OH)_2_]^+^ and [Au(C,N,N)(OH)_2_]^+^, the adsorption energies of −4 to −6 eV per molecule were obtained, while for the neutral [Au(C,N)(AcO)_2_] complex, the adsorption energy of -0.36 eV means a weaker, but still favorable adsorption. In addition, when an extra Cl^−^ anion is added to the external sphere of [Au(N,N)(OH)_2_]^+^ to make the system neutral, the adsorption energy in its flat-on coordination decreased significantly but remained negative (Table 1).

The charges obtained by Bader analysis [41] also confirm that a significant transfer of electrons occurs from the AuNPs surface to the positively charged complexes. However, the amount of transferred charge is almost equal for complex cations ([Au(N,N)(OH)_2_]^+^ and [Au(C,N,N,)(OH)_2_]^+^) but much lower (approaching zero) in the case of neutral [Au(C,N)(AcO)_2_] complex. Moreover, adsorption of positively charged species is further facilitated by the presence of the negative charge on the AuNPs surface, finally resulting in the neutralization of charge and the reduction of surface potential. Interestingly, when an extra Cl^−^ ion is added to the external sphere of [Au(N,N)(OH)_2_]^+^, the charge is transferred in the opposite direction from the absorption to the Au(Ш) surface.

Performed DFT calculations, in general, predict a fundamental difference between the interaction of charged and neutral complexes with the Au(Ш) model surface. The obtained result also points to the significance of the electrostatic interactions between the species on the AuNP surface, which is normally covered by citrate ions. The positively charged complexes in co-adsorption with citrates are expected to neutralize the AuNP surface charge and accelerate AuNPs agglomeration. On the other hand, the neutral complex exhibits weaker adsorption and does not burden AuNP with any charge. If co-adsorbed with citrate anions, [Au(C,N)(AcO)_2_] is not expected to affect AuNPs stability.

### 2.2. Spectrophotometric Study of the Interaction Between Gold(III) Complexes and AuNPs

**Figure 2:**
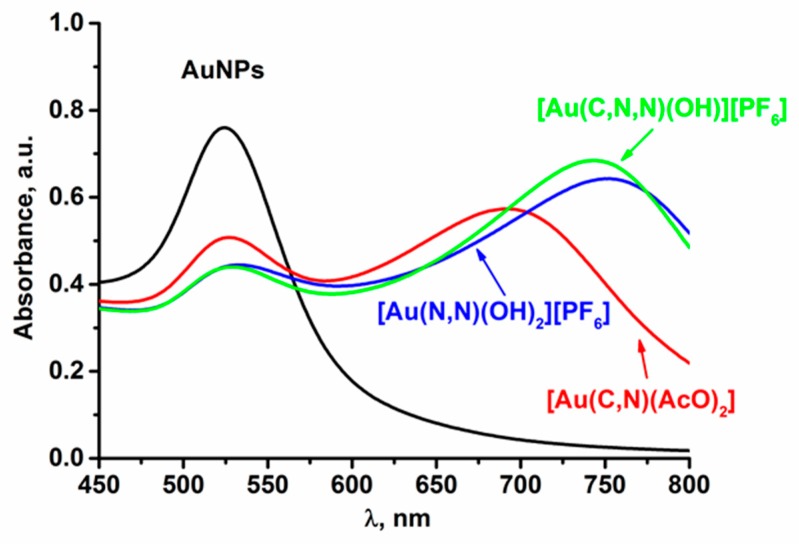
UV/Vis spectra of AuNPs/complex assembly containing 2 × 10^−10^ M AuNPs and 2 × 10^−6^ M gold(III) complexes recorded 60 min after preparing.

Since DFT calculations predicted the formation of stable nanocomposites between AuNPs and selected complexes, their interaction was further investigated by following the changes in the shape and position of the localized surface plasmon resonance (LSPR) band. The absorption spectra were followed after mixing the colloidal dispersion and complex solutions. In these experiments, the AuNPs concentration was 2 × 10^−10^ M, and the concentration of complexes was 2 × 10^−6^ M in all cases. The citrate-capped AuNPs have the characteristic LSPR band with one single peak at about 525 nm (Figure 2), which is characteristic for AuNPs having a diameter of ~30 nm [42].

It was found that the formation of AuNPs/complex nanocomposites induced the aggregation of functionalized AuNPs since the absorption spectra of colloid dispersion showed two absorption maxima. The appearance of a second peak is attributed mainly to the existence of linked NPs or larger aggregates due to the change of the interspacing distance between AuNPs [43]. As the interparticle spacing decreased, the first peak became weaker while the second peak gained intensity and shifted to longer wavelengths. The maximum peak shift was observed at the interparticle distance near zero, at which point the electrodynamic interaction between the NPs is at a maximum. The aggregation preceded through some time-dependent reaction steps [44]. In these processes, the complexes showed a slightly variable behavior, characterized by a continuous red shifting of the second LSPR maximum to 730–750 nm. This result indicates that SERS spectra of studied complexes should be effectively enhanced by AuNPs at the excitation wavelengths close to 730–750 nm due to the overlapping with the plasmon bands. The stabilization of the assembly was achieved within 60 min, followed further by precipitation within the next 24 h.

### 2.3. AFM Images of AuNPs /Au(III) Complexes Assembly

**Figure 3:**
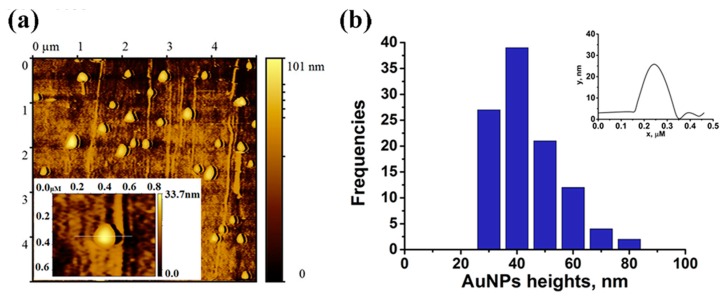
(**a**) AFM images in the topography mode of the AuNPs stabilized by citrate ions. Inset: isolated nanosphere. (**b**) Histogram of PSD. Inset: single AuNPs profile.

The AFM was applied to characterize the morphology, single AuNP profile, and the particle size distribution (PSD) of AuNPs functionalized by selected gold(III) complexes [45,46]. The results obtained for naked NPs clearly show that the isolated AuNP is spherical in shape with an overall particle size of 34 ± 5 nm (Figure 3).

**Figure 4:**
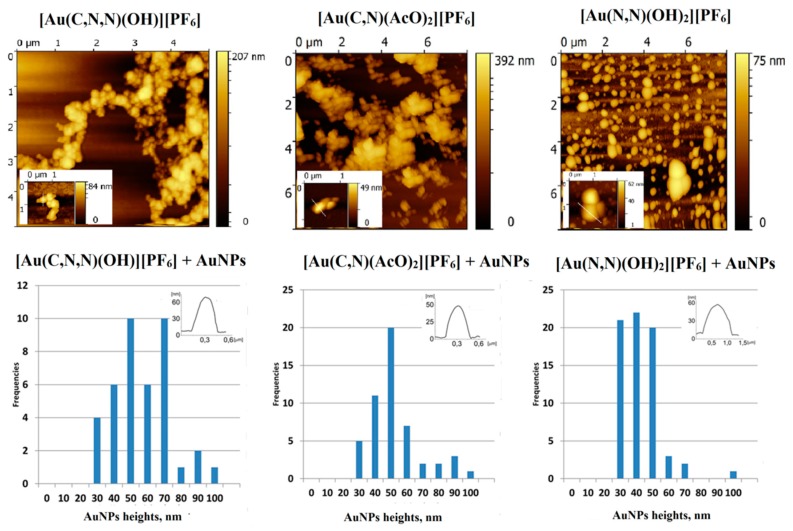
AFM images and histograms of size distribution of AuNPs in the presence of [Au(C,N,N)(OH)][PF_6_], [Au(C,N)(AcO)_2_] and [Au(N,N)(OH)_2_][PF_6_] in 2D topography. Insets: isolated aggregates and profiles of functionalized NPs.

The analysis of AFM images of AuNPs obtained after mixing with gold(III) complexes (Figure 4) suggested the significant changes in the particle morphology and PSD. The aggregates containing several NPs can be clearly seen in the insets of Figure 4. They are further linked forming string-like structures, as can be seen in the case of [Au(C,N,N)(OH)][PF_6_] and [Au(C,N)(AcO)_2_], or simply approach to each other forming plate-like structure as in the case of [Au(N,N)(OH)_2_][PF_6_]. However, it seems that the PSD of NPs in all structures increased due to the increase of radius of AuNPs-complex composite. This finding is in good agreement with spectrophotometric results and with the previously obtained TEM images [44] of complex/AuNPs conjugates. The results also confirm the assumption obtained by DFT calculations that complexes interact with citrate-stabilized AuNPs and adsorb on their surface, which corresponds to the determined PSD according to the literature [47,48].

### 2.4. SERS Spectra of Au(III) Complexes

**Figure 5:**
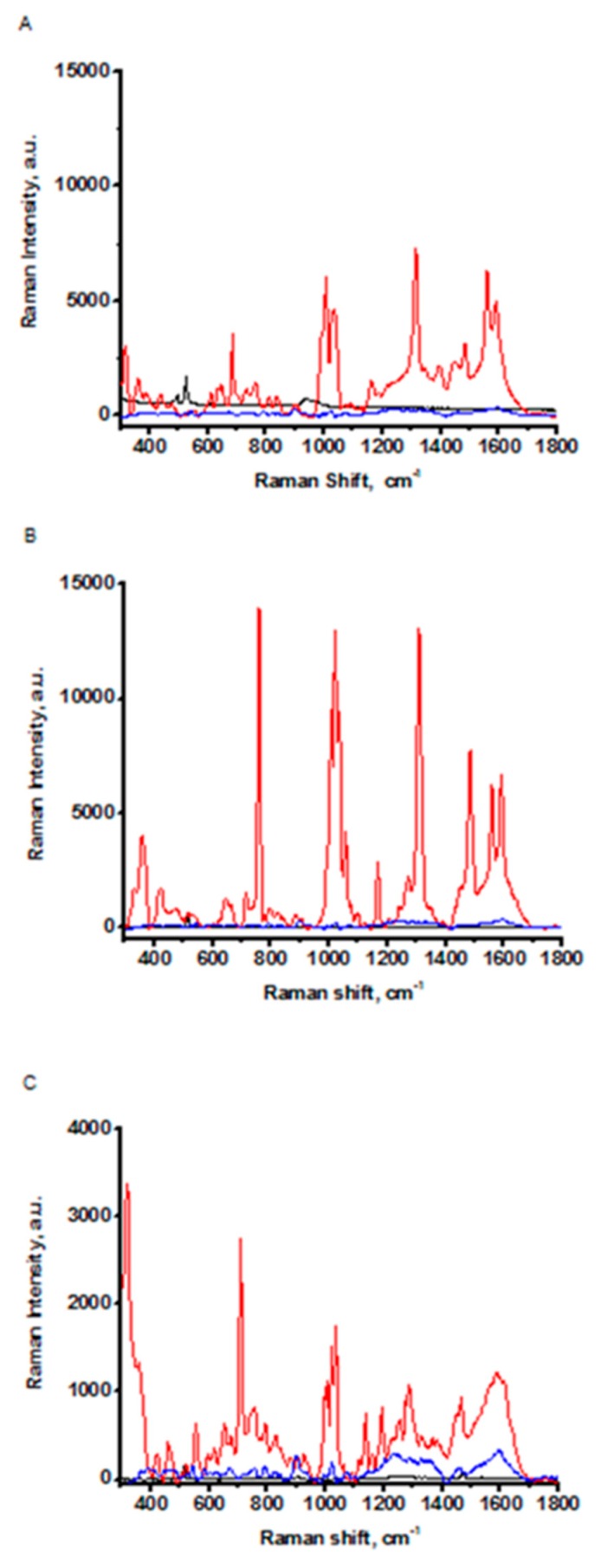
Raman spectra of 2 × 10^−6^ M Au(III) complexes (**A**) [Au(C,N,N)(OH)][PF_6_]; (**B**) [Au(N,N)(OH)_2_][PF_6_]; (**C**) [Au(C,N)(AcO)_2_] in the absence (black lines) and the presence (red lines) of 2 × 10^−10^ M AuNPs. The blue lines represent Raman spectra of AuNPs colloid.

To investigate the SERS effect of gold(III) complexes for potential biosensing applications, the Raman spectra of their 2 × 10^−6^ M solutions were followed in the absence and presence of 2 × 10^−10^ M AuNPs. The intensive resonant behavior was consistently observed for all studied complexes for excitation at 785 nm, close to the SPR band of nanocomposites. The concentration of the complexes was chosen in such a way that the surface of NPs was fully covered for studied particle size. The results (Figure 5) indicate the enhancement of SERS signals intensity in the range from 300–1800 cm^−1^. It is worthy to notice that the main marker bands of all nanocomposites correspond to marker bands of 2,2′-bipyridine and pyridine adsorbed on AuNPs particles [49,50,51]. For comparison and identification of peaks, the Raman spectra of powdered complex samples, and AuNPs colloid were also recorded and are presented in Supplemental materials (Figure S1). The Raman shifts of powdered complexes and their SERS shifts in the presence of AuNPs are given in Table 2, together with the Raman and SERS shifts of 2,2′-bipyridine and pyridine [49,50,51].

The SERS spectra of [Au(C,N,N)(OH)][PF_6_] and [Au(N,N)(OH)_2_][PF_6_] have the characteristic marker bands which are in accordance with the Raman spectra of bipyridine adsorbed on citrate capped AuNPs [52]. The SERS shifts can be in general divided into the aromatic ring stretching region (C–C, C–N, and C–H vibrations) between 1300 and 1650 cm^−1^, the ring breathing and C–H deformations at about 1000‒1180 cm^−1^ and the vibration of significant signal intensity at 755 cm^−1^ ascribed to in-plane ring deformation [51]. The shifts below 500 cm^−1^ correspond to Au–N stretches [53]. The analysis of SERS spectra suggests that the intensity of the signal for both complexes at about 1586 cm^−1^ appeared to be extraordinarily enhanced compared to SERS of bipyridine adsorbed on AuNPs [52]. This difference could be explained by considering the fact that the vibration of 2,2′-bipyridine is assumed to occur quite parallel to the metal surface. On the contrary, the conclusion can be made that the vibration of complexes does not occur in a quite parallel arrangement of the complex ion to the metal surface, as in the case of 2,2′-bipyridine. This is in accordance with the theoretically predicted tilt binding geometry.

SERS spectra of [Au(C,N)(AcO)_2_] have some shifts characteristic for SERS of pyridine on the Au coated metal surface [49,54]. However, the intensity of peaks in-ring breathing and C-H stretching region (1000–1180 cm^−1^) and aromatic ring stretching region (1597 cm^−1^ and 1639 cm^−1^) is lower compared to ring deformation in the plane and shifted from 636 cm^−1^ to 707 cm^−1^. This is not characteristic for the SERS spectra of the other two complexes.

### 2.5. AFM and SERS Tracking of Gold(III) Complexes in the Cytosolic, Nuclear, and Plasma Membrane Fractions of Human Lymphocytes

In order to obtain further insight into the application of AFM and SERS methods for biosensing purposes [30,55], the study of the intracellular trafficking of selected complexes in the living cells was performed. The lymphocytes were treated with the investigated gold(III) complexes and their cytosolic, nuclear and membrane fractions were separated before and after the treatment. All fractions were subjected to AFM and Raman analysis in order to qualify the sub-cellular distribution of complexes in cytosolic, nuclear and membrane fractions.

**Figure 6:**
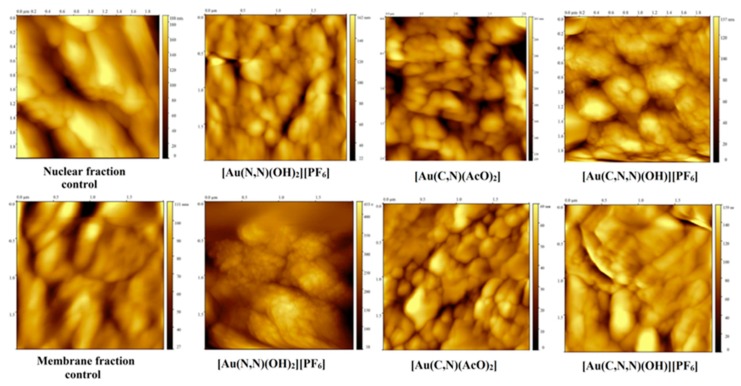
AFM images of nuclear and membrane fractions (control) and in the presence (right) of gold(III) complexes.

The AFM images of nuclear and membrane cell fractions before and after the treatment of cells with the investigated complexes are presented in Figure 6.

The typical image of nuclear fraction patches shows the string-like structure well distributed on the surface. However, upon the treatment of lymphocytes with the investigated complexes, the nuclear morphology changed significantly. The height of the patches decreased, and the fragments became smaller and damaged with several cracks and holes. Most of the material was divided into smaller spherical aggregates.

Furthermore, all investigated complexes induced changes in morphology and nanostructure of the membrane fraction. The [Au(C,N)(AcO)_2_] complex induced changes similar to those observed in the nuclear fraction while the other two complexes induced the swelling of the membrane fragments (Figure 6). These results imply that the presence of gold(III) complexes caused nuclear and membrane changes on a nanoscale, resulting in a greater effective membrane surface.

**Figure 7:**
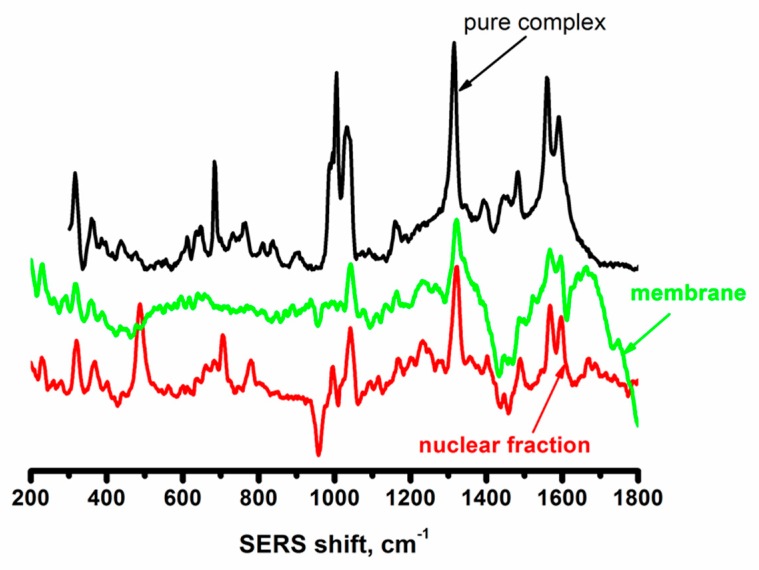
SERS spectra [Au(C,N,N)(OH)][PF_6_] in water, nuclear, and membrane fractions. The spectra are intensity-shifted for comparison.

SERS analysis of all cell fractions was performed after the samples were briefly incubated with 2 × 10^−10^ M dispersion of AuNPs. As an example, the corresponding SERS spectra of [Au(C,N,N)(OH)][PF_6_] in the membrane and nuclear fractions are shown in Figure 7, together with the SERS spectrum of the pure complex. The SERS shift positions are carefully analyzed in order to compare them with the pure complexes and are presented in Table 2. However, in the nuclear and membrane fractions, [Au(C,N,N)(OH)][PF_6_] displayed the characteristic SERS vibrations with the significant intensive marker bands. These bands mainly originate from the ligand, i.e., 2,2′-bipyridine, as also found by SERS spectra of the pure complex in the presence of AuNPs. This finding is also supported by an inspection of the data presented in Table 2. In addition, the extensive biological matrix present in each sample did not affect the positions of marker bands significantly. On the contrary, it affected the intensity ratio of some of the distinct marker bands.

It must be pointed out that the complex was not observed in the cytosol fraction. This was also found for the other two complexes, [Au(N,N)(OH)_2_][PF_6_] and [Au(C,N)(AcO)_2_]. The SERS spectra of these two complexes in water, as well as in the nuclear and membrane fractions, are given in the Supplementary Materials (Figure S2), and the bands’ positions are also listed in Table 2. In the case of [Au(N,N)(OH)_2_][PF_6_] the marker bands can be identified in the nuclear fraction, but in the case of [Au(C,N)(AcO)_2_] the signals of both fractions have the intensity slightly above the noise. It is also worthily to notice that this complex has the Raman spectra of the powdered compound and also SERS with low bands intensity compared to the other two complexes. Besides, it seems that the biological matrix affected the intensity rations of SERS signals of [Au(N,N)(OH)_2_][PF_6_] so that the presence of this complex in cell fractions cannot be fully confirmed. Based on this findings it seems that SERS method is the conventional technique for tracking of gold(III) complexes in the tissue, but the sensitivity of the method is limited for some complexes due to the low signal intensity of Raman spectra, as in the case of [Au(C,N)(AcO)_2_]. However, AFM images of nuclear and cell fractions after the cell treatment with all selected complexes suggest that the complexes passed the membrane and reached the nuclear fraction in both cases.

## 3. Discussion

With the increasing interest in the synthesis of new gold(III) complexes with pyridine and 2,2′-bipyridine ligands having potential antitumor activity, there is also the growing need for tracking of their fate in biological tissues. In parallel, AuNPs represent an excellent nanoplatform in developing analytical methods for biosensing or drug delivery, but there is a limited number of literature data that consider AuNPs as carriers for antitumor metal complexes [23,29,58]. Moreover, there is the possibility to use AuNPs functionalized by these complexes for applications as drug delivery nanocomposites [58,59]. DFT studies of optimal binding structures to Au(Ш) surface predicted the formation of stable nanocomposites in the case of all studied complexes, offering the tilt binding geometry on the AuNPs surface. This conclusion is in accordance with previous findings concerning the adsorption of their ligands (2,2′-bipyridine and pyridine) and corresponding derivatives on the Au(Ш) surface [51,56,57,60], which suggest their parallel or vertical orientation on AuNPs. This orientation is strongly influenced by the occupation of orbitals in the pyridine ring due to the protonation or coordination of N atoms. According to the literature data, 2,2′-bipyridine is adsorbed on Au(Ш) surface with the two aromatic rings parallel (flat-on coordination) to the negatively charged gold surface [51,61], or by overlapping of Au d orbitals with lone electron pairs of two N atoms in vertical (end-on conformation). Moreover, Zn and Ru complexes of 2.2′-bipyridine indicated cisoid (twisted)-conformation on the negative Au electrode surface. It is also worthily to notice that for pyridine derivatives, the orientation on Au(Ш) surface also changed from vertical to parallel [62].

In the case of the studied complexes, nitrogen atoms are coordinated by gold(III) forming square planar complexes. DFT calculations indicated that the adsorption energy due to the interaction of complex cation via oxygen of hydroxyl and acetyl groups (end-on conformation) is significantly lower compared to the physisorption in flat-on conformation. The stability of the obtained nanocomposites, confirmed spectrophotometrically in our previous study [44], is the consequence of a high degree of affinity, i.e., adsorption energy, between the positively charged complexes and predominantly negatively charged AuNPs. However, a transition between flat-on and end-on binding seems realistic [57], resulting in tilt complex orientation on the AuNPs surface.

The red shifting of the SPR bands position from visible to near-infrared region indicates that the aggregation of NPs occurred and that this process is very similar for all three complexes. Moreover, NPs aggregation occurred also due to the adsorption of 2,2′-bipyridine ligand on citrate capped AuNPs followed by the spectral changes in the same wavelength range [56]. However, SERS spectra of 2,2′-bipyridine and pyridine derivatives adsorbed on the Au(Ш) surface [49,51,56] are consistent with the marker bands obtained for pure complexes investigated in the present paper. It can be noticed that the SERS of investigated complexes has slightly shifted bands with enhanced intensity. A similar behavior was also observed for Raman spectra of complexes AubipyCl_3_, ZnbipyCl_2_, and Ru(bipy)_2_ Cl_2_ in solid-state and also solution [51,53]. Moreover, we assume that the vibrations with strongly enhanced intensities in the range below 500 cm^−1^ are carrying the information on the complex-NPs interaction. These shifts belong to the vibrations of the N–Au–O bond in [Au(N,N)(OH)_2_][PF_6_] and [Au(C,N,N)(OH)][PF_6_]. For [Au(C,N)(AcO)_2_], this information is based on the N–Au–O–C–O bond between acetyl group and AuNPs [63].

The AFM is a powerful nanotechnology tool that has been applied to observe nanostructural details and biomechanical properties of cell extracts for obtaining the information of ligand/receptor interaction [27,30,64]. In our experiments, the ultra-high force sensitivity of the AFM and its ability to measure nanomechanical properties of individual cells or their parts indicated the changes in the morphology of all studied cell extracts near physiological conditions. The AFM images undoubtedly confirmed that the morphology and nanostructure of cell fractions (except the cytosol) changed significantly under the influence of all complexes.

These findings suggested that the studied complexes diffused across the cell membrane and reached the nucleus, inducing the change of cell fraction morphology and nanostructure in all cases. However, these changes are in accordance with the inhibition of the activity of some enzymes, i.e., Na/K-ATPase, as shown in our previous studies [17,18,19]. The conclusion can be made that the complexes targeted the protein structure. The presence of any of marker bands in cellular extracts can confirm the presence of gold(III) complexes in related cellular fractions. Based on the intensity of the marker bands and the position of SERS shifts, the conclusion can be made that only [Au(C,N,N)(OH)][PF_6_] was detected with a significant presence in both membrane and nuclear cell fractions. It seems that [Au(C,N,N)(OH)][PF_6_] was found unaltered in these fractions, as also found previously for some drugs [22,25,65,66]. However, low-intensity SERS signals with some of the characteristic shifts can also be recognized for the other two complexes. Assuming that the complexes reach all cell fractions, there are two possible reasons for a decrease in their concentration. One is variable kinetics of their uptake across the plasma membrane. The other is an increase in the rate of drug efflux through the membrane to the cell nucleus. However, our findings indicate that the complexes passed the membrane, reached the nuclear fraction, and exited the cell fraction with various kinetics. Their fate during this traffic cannot be clarified based on the results of our experiments. However, the comparison can be made with the behavior of *cis*-Pt [11] and its transformation to side products without the significant retention in cytoplasm fraction. It is shown that neutral cisplatin is capable of passively diffusing across a lipid bilayer into the cytoplasm of cells, where it accumulates under the physiological conditions as the positively charged di-aqua derivative due to hydrolysis, which cannot leave the cell, inducing thus the toxic effects of drugs.

## 4. Materials and Methods

### 4.1. Chemicals

Gold(III) chloride trihydrate (HAuCl_4_ × 3H_2_O), trisodium citrate dihydrate (C_6_H_5_Na_3_O_7_ × 2H_2_O), and dimethyl sulfoxide DMSO were purchased by Sigma (St. Louis, MO, USA). [Au(C,N,N)(OH)][PF_6_], [Au(N,N)(OH)_2_][PF_6_], and [Au(C,N)(AcO)_2_] were synthesized as described in the literature [3,12]. The terdentate C,N,N ligand arises from 6-(1,1-dimethylbenzyl)-2,2′-bipyridine, whereas the bidentate C,N and N,N ligands arise from deprotonated 6-(1,1-dimethylbenzyl)-pyridine and 2,2′-bipyridine, respectively.

1 × 10^−2^ M stock solutions of complexes in DMSO were prepared shortly before use. Working solutions were prepared by diluting the stock solutions with deionized water to desired concentrations.

### 4.2. Synthesis of AuNPs

The colloid suspension of AuNPs was synthesized by a modified Turkevich method [31] as previously described using sodium citrate as a reducing and a loosely bound capping agent [32]. According to this procedure, a 200 mL aqua solution of HAuCl_4_ (1 mM) was vigorously stirred and heated in a round-bottom flask fitted with a reflux condenser. Then, 10 mL of 38.8 mM sodium citrate was rapidly added to the boiling solution and was kept boiling for another 15 min, when its color changes from pale yellow to wine-red. The resulting 0.95 mM colloid solution was stable for several months at 4 °C. The concentration of colloid dispersion was calculated using the following equation:

(1) $$c=\frac{A_{r}c_{M}}{\frac{4}{3}r^{3}\pi \rho N_{o}}$$

where *A_r_* represents relative atomic weight of gold, *C_M_* gold(III) concentration in working solution, *r* nanoparticles diameter, *ρ* density of gold atom and *N_o_* Avogadro constant.

### 4.3. Subcellular Fractionation of Human Lymphocytes

The blood sample (25 mL) was obtained from a healthy volunteer in accordance with the current Health and Ethical regulations. Fresh human lymphocytes were isolated using Histopaque density gradients purchased from Sigma (St. Louis, MO, USA), following the protocol of the manufacturer. After washing in phosphate buffer solution (PBS), the lymphocytes were placed in cultures containing PB-max karyotyping medium and incubated at 37 °C for 72 h. After washing in PBS, four parallel sets of lymphocyte cultures were set up (untreated control and samples treated with selected Au(III) complexes) and were incubated for the next 30 min at 37 °C. After washing in PBS, the lymphocytes were kept in 2 mL of hypotonic buffer (42 mM KCl, 10 mM 4-(2-hydroxyethyl)-1-piperazineethanesulfonic acid (HEPES, pH 7.4) and 5 mM MgCl_2_) for 15 min at 4 °C. Afterward, the cells were passed through a 30 gauge needle 10 times. The extract was centrifuged at 250× *g* for 10 min to remove the nuclei and intact cells. The postnuclear supernatant (PNS) was centrifuged at 150,000× *g* for 30 min at 4 °C to separate the cytoplasm from the membrane fraction. The cellular compartments were stored under controlled conditions (−80 °C) until analysis.

### 4.4. Apparatus

UV/Vis spectra were recorded on Lambda 35 UV-Vis Spectrometer, (Perkin Elmer, Shelton, CT, USA) with a thermostated 1.00 cm quartz cell at 25 °C. All spectra were background-subtracted against deionized water, which is reaction solvent. The spectra were recorded in the function of the time immediately after the addition of the appropriate volume of gold complex stock solution into 2 × 10^−10^ M AuNPs dispersion.

AFM (home made, Consiglio Nazionale delle Ricerche, Roma, Italy) [33] measurements were performed in the air (at room temperature), with the microscope working in non-contact (tapping mode) using AFM instrument with NT-MDT silicon NSG30 cantilevers described elsewhere [33]. The tips with an expected statistical apical radius of 10 nm were 125 µm long with resonant frequencies typical of 320 kHz and a constant force typical of 40 N/m. The drop of the sample was put on the glass surface and dried at 37 °C for one hour. Constant and lateral force images have been acquired simultaneously at a set point of 50% from free oscillation with a typical scan rate of 2.0–4.0 sec/row (400–800 points/row). The lateral friction images (not shown) have been found very powerful because of their sensitivity to the small structures that protrude from a large corrugated surface. Data have been treated by only a background subtraction and then analyzed using the Gwiddion software.

The Raman spectra were acquired on an InVia spectrometer (Renishaw LTD, London, United Kingdom) from Renishaw LTD equipped with a solid-state laser source emitting at 785 nm. A 50 X, 0.48 NA long working distance microscope objective was used for illumination and collection in backscattering configuration. Acquisition time was set at 10 s with 5 accumulations per spectrum. Laser power at the focus was set to 5% (with 100% = 300 mW). A drop of solution (~20 µL) for each preparation was put on a silicon substrate and then stored for 30 min in a warm box in order to dry the solution. An air-cooled 785 nm compact laser system (CLS) high brightness laser optimized for micron resolved measurements with an output power of 84 mW was used to acquire spectra. The acquisition time for each spectrum was 10 s averaged over three iterations, with the subsequent removal of cosmic rays.

### 4.5. DFT Calculations

Calculations were performed using the pWscf code of Quantum ESPRESSO computational package for electronic-structure calculations and materials modeling [34]. Exchange-correlation functional in Generalized gradient approximation of Perdew, Burke and Erzenhorf (GGA-PBE) [35], and ultrasoft pseudopotentials with Gaussian smearing were used. Kinetic energy cutoff was set to 25 Ry, while the charge density cutoff was 200 Ry. The AuNP surface was modeled as a three-layer Au(Ш) slab (16 Au atoms per layer), in a 44 supercell. The bottom layer was fixed, and the two upper layers were allowed to relax. The vacuum thickness between slabs was fixed to 70 Bohr. The first irreducible Brillouin zone was sampled by a 2 × 2 × 1 set of k-points generated through the general Monkhorst–Pack scheme [36]. The electronic convergence criterion was set to 10^−5^ eV, and the force convergence threshold for ionic minimization in geometry optimization was set to 1.5  × 10^−4^ eV/Å. All calculations were spin restricted. For the graphical representation of surface models XCrysDen program for crystalline and molecular structure vizualisation [37] was used. Adsorption energies were calculated as:

(2) $$E_{ads}=E_{mol+surf}-E_{mol}-E_{surf}$$

where *E_mol + surf_* is a DFT calculated as the total energy of the geometrically optimized surface with an adsorbed Au-complex molecule, *E_mol_* is the DFT of the total energy of the isolated Au-complex in the same supercell and *E_surf_* is the DFT total energy of the Au(Ш) surface slab.

Charged systems were modeled through the addition of a single positive charge to a supercell, which was compensated by a uniform jellium background as implemented in Quantum ESPRESSO. Charge analysis was performed using Bader code [38]. Semiempirical Van der Waals correction DFT + D2 was applied according to Grimme [39].

## 5. Conclusions

The functionalization of 30 nm citrate-capped AuNPs by the series of mononuclear antitumor gold(III) complexes ([Au(C,N,N)(OH)][PF_6_], [Au(N,N)(OH)_2_][PF_6_] and [Au(C,N)(AcO)_2_]) led to the formation of stable nanostructured assemblies that allow the tracking of their subcellular distribution in cell fractions using the AFM and SERS techniques. The formation of gold(III) complex/AuNPs conjugates, and their physicochemical properties were characterized by various methods (DFT calculations, UV/Vis spectrophotometry, AFM, Raman spectroscopy, and SERS). The stable spherical conjugates linked in a string- or plate-like structures showed a dramatic resonance enhancement in Raman spectra induced by AuNPs, both in aqua solution, as well as in membrane and nuclear fractions of the cell. To test the application of functionalized AuNPs for biosensing, the distribution of investigated complexes in nuclear, cytosolic and membrane cell fractions obtained from human lymphocytes was studied by AFM and SERS measurements. No complex was detected in the cytosolic fraction, as revealed by SERS measurements. [Au(C,N,N)(OH)][PF_6_] was detected in the membrane and nuclear cell fractions. [Au(N,N)(OH)_2_][PF_6_] was detected in the nuclear fraction, but both [Au(N,N)(OH)_2_][PF_6_] and [Au(C,N)(AcO)_2_] were not detected in membrane fractions with the significant reliability. The AFM method confirmed that the complexes induced changes in the morphology and nanostructures of the membrane and nuclear fractions. In general, the obtained results indicate that the formed conjugates of AuNPs and selected gold(III) complexes could be a tool for the detection of the drug distribution in cell fractions.

## Figures and Tables

**Table 1:** Results of DFT calculations for interaction between AuNPs and gold(III) complexes.

Complex + Surface	Closest Complex-Surface Distance (Å)	E_ads_ (eV)	Charge Received by Complex Molecule (e)	Optimized Binding Geometry
[Au(N,N)(OH)_2_]^+^ + Au111 (*parallel*)	3.27	−6.09	−0.118	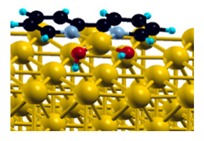
[Au(N,N)(OH)_2_]^+^ + Au111 (*vertical*)	2.55	−4.07	−0.097	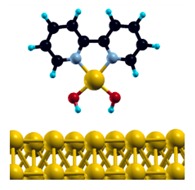
[Au(N,N)(OH)_2_]^+^Cl + Au111	3.88	−0.43	−0.231	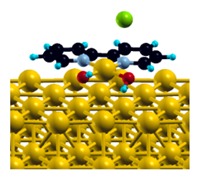
[Au(C,N)(AcO)_2_] + Au111	2.83	−0.36	−0.012	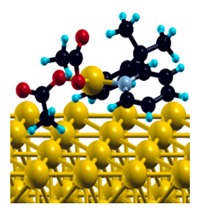
[Au(C,N,N)(OH)]^+^ + Au111	2.13	−4.48	−0.110	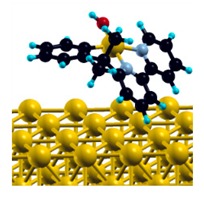

**Table 2:** Tentative assignments of Raman shifts of selected complexes and SERS signals of their AuNPs composites in the cell membrane and nuclear fractions.

Complex	Assignment ^a^	Raman Vibrational Mode ^a^ (cm^−1^)	SERS Vibrational Mode (cm^−1^)	Membrane Fraction (cm^−1^)	Nuclear Fraction (cm^−1^)
[Au(C,N,N)(OH)] [PF_6_]	Au–N stretching	324	316	321	321
		602	602	610	−
	In-plane ring deformation	741	756	−	778
	C–H ring breathing	1049	1031	1040	1043
	Aromatic C–C stretching	1327	1315	1319	1319
		1496	1484	1486	1487
		1575	1559	1568	1568
		1603	1592	1591	1601
[Au(C,N)(AcO)_2_]	Au–N stretching	317	319		
		−	361		
		−	460		
		523	549		
		660	652		
	In-plane ring deformation	−	758		
	C–H ring breathing	1046	1037		
		1272	1270		
	Aromatic C–C stretching	−	1592		
			1616		
[Au(N,N)(OH)_2_] [PF_6_]	Au–N stretching	342	357		342
		475	421		482
		725	−		−
	Ring deformation in the plane	757	757		707
		882	1006		996
		−	1021		1026
	C–H ring breathing	1051	1055		1168
		1326	1308		1315
		1504	1484		1443
	Aromatic C–C stretching	1572	1559		1547
		1609	1592		1608
2,2′-bipyridine ^b^	Out-of-plane ring deformation	328	354		
		548	−		
		611	646		
	In-plane ring deformation	752	761		
		992	1010		
		1043	1057		
	C–H ring breathing	1298	1301		
		1480	1479		
		1570	1562		
	Aromatic C–C stretching	1588	1586		
Pyridine ^b^	Out-of-plane ring deformation		389		
		385			
	In-plane ring deformation	618	636		
	C–H ring breathing	1002	1009		
		1036	1035		
		1220	1213		
	Aromatic C–C stretching	1583	1597		
		-	1639		

^a^ Solid state spectra, ^b^ Assignments are derived from references [49,51,56,57].

## Supplementary Materials

The following are available online at https://www.mdpi.com/1422-0067/20/24/6306/s1, Figure S1: Comparison of Raman (black lines) and SERS (red lines) spectra of [Au(C,N,N)(OH)][PF_6_], [Au(N,N)(OH)_2_][PF_6_], and [Au(C,N)(AcO)_2_] complexes. c_complex_ = 2 × 10^−6^ M, cAuNPs = 2 × 10^−10^ M., Figure S2: SERS spectra of 2 × 10^−6^ M [Au(N,N)(OH)_2_][PF_6_], and [Au(C,N)(AcO)_2_] in water solution, nuclear, and membrane cell fractions, in the presence of 10^−10^ M AuNPs.

## Abbreviations

(C:N)	deprotonated 6-(1,1-dimethylbenzyl)-pyridine
(C,N,N)	deprotonated 6-(1,1-dimethylbenzyl)-2,2′-bipyridine
(N,N)	2,2′-bipyridine
PBS	Phosphate buffer solution
HEPES	4-(2-hydroxyethyl)-1-piperazineethanesulfonic acid
DFT	Density functional theory
CLS	Compact laser system
AFM	Atomic Force Microscopy
SERS	Surface-enhanced Raman spectroscopy
LSPR	Localized surface Plasmon resonance
NPs	Nanoparticles
AuNPs	Gold nanoparticles
AgNPs	Silver nanoparticles
