# Supplementary material for: Conjugates of Gold Nanoparticles and Antitumor Gold(III) Complexes as a Tool for Their AFM and SERS Detection in Biological Tissue
Source: Int J Mol Sci. 2019 Dec 13;20(24):6306. doi: 10.3390/ijms20246306 (PMC6940825; doi:10.3390/ijms20246306)
Supplement: Supplementary file 1 [file ijms-20-06306-s001.pdf]

Supplementary Materials

Conjugates of AuNPs and antitumor gold(III) complexes as a tool for their AFM and SERS detection in the biological tissue

Aleksandra M. Bondžić[1], Andreja R. Leskovac[1], Sandra Ž. Petrović[1], Dragana D. Vasić Anićijević[1], Marco Luce[2], Lara Massai[3], Amanda Generosi[2], Barbara Paci[2], Antonio Cricenti[2], Luigi Messori[3], Vesna M. Vasić[1*]

[1]*Vinča Institute of Nuclear Sciences, University of Belgrade, P.O. Box 522, 11000 Belgrade, Serbia*
[2]*Istituto di Struttura della Materia, Consiglio Nazionale delle Ricerche, Roma, Italy*
[3]*Department of Chemistry, University of Florence, Via della Lastruccia 3, 50019 Sesto Fiorentino, Italy*

**corresponding author: evasic@vin.bg.ac.rs*

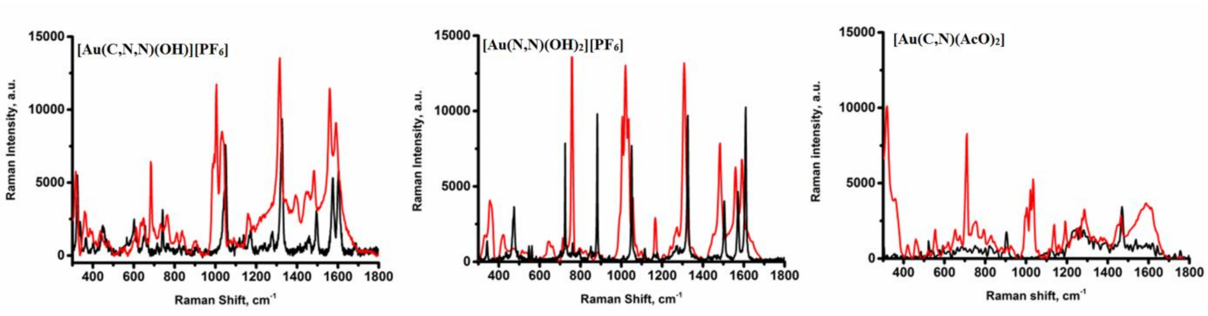

Figure S1. Comparison of Raman (black lines) and SERS (red lines) spectra of $[Au(C,N,N)(OH)][PF_6]$, $[Au(N,N)(OH)_2][PF_6]$ and $[Au(C,N)(AcO)_2]$ complexes. $c_{complex} = 2 \times 10^{-6}$ M, $c_{AuNPs} = 2 \times 10^{-10}$ M.

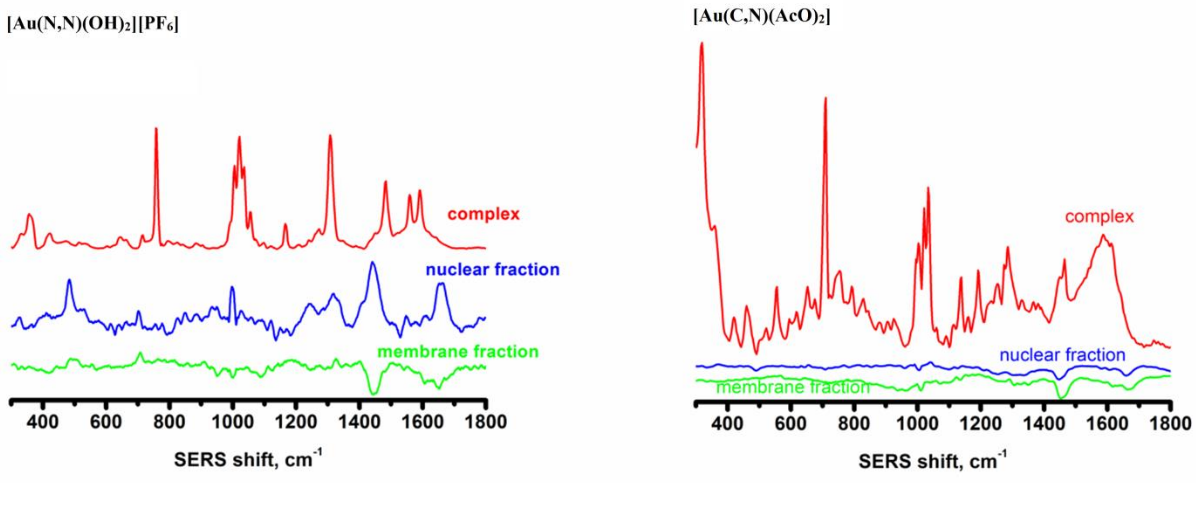

Figure S2. SERS spectra of 2×10^{-6} M $[Au(N,N)(OH)_2][PF_6]$ and $[Au(C,N)(AcO)_2]$ in water solution, nuclear and membrane cell fractions, in the presence of 10^{-10} M AuNPs.
